# Supplementary material for: Effects of diuretic administration on outcomes of extracorporeal shockwave lithotripsy: A systematic review and meta-analysis
Source: PLoS One. 2020 Mar 5;15(3):e0230059. doi: 10.1371/journal.pone.0230059 (PMC7058295; doi:10.1371/journal.pone.0230059)
Supplement: S1 Checklist — (DOC) [file pone.0230059.s001.doc]

| **Section/topic** | **#** | **Checklist item** | **Reported on page #** |
| --- | --- | --- | --- |
| **TITLE** | | |  |
| Title | 1 | Effects of Diuretic Administration on Outcomes of Extracorporeal Shockwave Lithotripsy: A Systematic Review and Meta-analysis | 1 |
| **ABSTRACT** | | |  |
| Structured summary | 2 | Background The present systematic review and meta-analysis of randomized controlled trials (RCTs) was conducted for investigating the effect of diuretics on the outcomes of shockwave lithotripsy (SWL) for the treatment of urinary stones.  Material and Methods We performed searches of PubMed, Web of science, Embase, EBSCO, and Cochrane library databases from inception to November 2019. RCTs were selected for assessing the effects of diuretics on fragmentation and clearance of urinary stones. The search strategy and study selection process were performed in accordance with the PRISMA guidelines.  Results Four RCTs were included in the meta-analysis. Overall, intervention groups experienced significant improvements in fragmentation compared with the control groups (risk ratio [RR] = 1.14, 95% confidence interval [CI] = 1.05–1.03, P = 0.02). However, stone clearance did not significantly differ between the intervention and control groups (RR = 1.23, 95% CI = 0.97–1.56, P = 0.08). The total numbers of shocks and sessions required were significantly reduced by the use of diuretics.  Conclusion Diuretics significantly enhance stone fragmentation for patients undergoing SWL. However, the improvement in stone clearance appears to be insignificant | 2 |
| **INTRODUCTION** | | |  |
| Rationale | 3 | Adjuvant interventions are urgently required for improving the results of SWL in terms of residual stone removal and overall efficacy. For reducing the necessity of more invasive treatments such as ureteroscopy, less-invasive interventions including inversion therapy, mechanical percussion, and drug therapy have been explored. Among these, pharmacotherapy is considered as a promising approach, with medicines such as calcium channel blockers, α-adrenergic blockers, nonsteroidal anti-inflammatory drugs, and progesterone being proven to have beneficial effects on the expulsion of stones and efficacy of SWL. However, the effects of diuretics on the success of SWL remain unclear from the present literature. | 3 |
| Objectives | 4 | We performed a systematic review and meta-analysis of randomized controlled trials (RCTs) for investigating the effects of diuretic administration during SWL on outcomes. | 4 |
| **METHODS** | | |  |
| Protocol and registration | 5 | This study is not registered. | - |
| Eligibility criteria | 6 | (1) RCT study design, (2) the intervention was SWL with the use of diuretics versus SWL with placebo (or with no intervention), (3) adequate reporting of data provided for analysis, and (4) availability of the entire text. | 4-5 |
| Information sources | 7 | We systematically searched several databases including PubMed, EMbase, Web of science, EBSCO, and the Cochrane library from inception to November 2019 with the following keywords: “diuretic,” “shock wave lithotripsy,” “furosemide,” “drug therapy,” and “urolithiasis.” | 5 |
| Search | 8 | The article selection process was performed in accordance with the PRISMA guidelines. | 5 |
| Study selection | 9 | The reference lists of retrieved studies and relevant reviews were hand-searched, and the process mentioned above was repeatedly performed for ensuring that all eligible studies were included. | 5 |
| Data collection process | 10 | Data were independently extracted by two investigators (W.Z.H and B.Y.J). Discrepancies were resolved by consensus. | 5 |
| Data items | 11 | We assess Standard Mean differences (Std. MDs) with 95% confidence intervals (CIs) for continuous outcomes, and risk ratios (RR) with 95% CIs for dichotomous outcomes. Heterogeneity is evaluated using the I^2^ statistic, and I2 > 50% indicates significant heterogeneity. | 5 |
| Risk of bias in individual studies | 12 | We used the Jadad Scale to evaluate the quality of the study. | - |
| Summary measures | 13 | Risk ratios (RR) with 95% confidence intervals (CIs) were calculated for dichotomous outcomes. Heterogeneity was evaluated using the I2 statistic | 5 |
| Synthesis of results | 14 | I2 > 50% taken to indicate significant heterogeneity. | 5 |

Page 1 of 2

| **Section/topic** | **#** | **Checklist item** | **Reported on page #** |
| --- | --- | --- | --- |
| Risk of bias across studies | 15 | The Jadad scores of the included studies varied from two to four. One study was considered to be low quality while other three studies were considered to be high quality. | - |
| Additional analyses | 16 | Sensitivity analysis was performed for evaluating the influence of a single study on the overall estimate by omitting one study in turn or performing subgroup analysis. | 5 |
| **RESULTS** | | |  |
| Study selection | 17 | A total of 389 articles were initially identified from database searches. After the removal of duplicates, 255 articles were retained. Of these, 247 were excluded from analysis following the screening of the abstracts and titles, three were excluded due to study design, and one was excluded because of insufficient data. Four RCTs were identified for satisfying the inclusion criteria, and they were finally enrolled in this meta-analysis. | 6 |
| Study characteristics | 18 | Shown in the table 1. | 7 |
| Risk of bias within studies | 19 | - | - |
| Results of individual studies | 20 | Shown in the table 1. | 7 |
| Synthesis of results | 21 | A random-effects model was used for analyzing the primary outcomes. Compared with control groups, our results indicated that the use of diuretic significantly improved the fragmentation achieved by SWL (RR = 1.14, 95% CI = 1.05–1.03; P = 0.02) with insignificant heterogeneity among the studies (I2 = 0%, P = 0.42, Fig 2). Although the outcome of stone clearance showed some differences between the studies, this was not noted to be statistically significant (RR = 1.23, 95% CI = 0.97–1.56, P = 0.08) with significant heterogeneity (I2 = 74%, P = 0.01, Fig 3). The study by Sabharwal et al. reported mean total numbers of shocks in experimental and control groups of 3,661.4 ± 1,946 and 3,894.7 ± 2,254, respectively (P < 0.05), and a mean number of sessions of 2.12 ± 1.17 and 2.25 ± 1.3, respectively (P < 0.05). Zomorrodi et al. reported 5,300 and 6,293 shocks, respectively (P < 0.05) and 1.5 and 1.92 sessions, respectively (P < 0.05). The above results could not be subjected to meta-analysis due to incomplete data; however, the numbers of shocks and sessions were not significantly affected by the use of diuretic. | 7-8 |
| Risk of bias across studies | 22 | - | - |
| Additional analysis | 23 | Sensitivity analysis was performed to evaluate the stability of the results. After removing the study by Yoon et al., heterogeneity was low (I2 = 2%, P = 0.36) and the outcome of stone clearance remained statistically insignificant (RR = 1.11, 95% CI = 1.00–1.23, P = 0.02). | 8 |
| **DISCUSSION** | | |  |
| Summary of evidence | 24 | The use of diuretics during SWL improve fragmentation of urinary stones. Nevertheless，the improvement in stone clearance does not appear to be significant. | 9 |
| Limitations | 25 | First, there was insufficient data regarding adverse effects associated with the use of diuretics for further analysis. Second, differences in stone size, location, and type as well as the SWL machine which may cause unpredictable bias are worth considering in future studies. Lastly, some unpublished and missing negative data may result in bias toward the summary effect. | 10 |
| Conclusions | 26 | The use of diuretics appears to significantly improve stone fragmentation in patients undergoing SWL. However, the improvement in stone clearance does not appear to be significant. | 10 |
| **FUNDING** | | |  |
| Funding | 27 | N/A | - |

*From:*  Moher D, Liberati A, Tetzlaff J, Altman DG, The PRISMA Group (2009). Preferred Reporting Items for Systematic Reviews and Meta-Analyses: The PRISMA Statement. PLoS Med 6(7): e1000097. doi:10.1371/journal.pmed1000097

For more information, visit: **www.prisma-statement.org**.

Page 2 of 2
