# Supplementary material for: Effects of diuretic administration on outcomes of extracorporeal shockwave lithotripsy: A systematic review and meta-analysis
Source: PLoS One. 2020 Mar 5;15(3):e0230059. doi: 10.1371/journal.pone.0230059 (PMC7058295; doi:10.1371/journal.pone.0230059)
Supplement: Attachment — Submitted filename: Response to Reviewers.docx [file pone.0230059.s002.docx]

**Dear Editor**

**Thank you very much for giving us an opportunity to revise our manuscript. We appreciate the editor and reviewers very much for their constructive comments and suggestions on our manuscript entitled “Effects of Diuretic Administration on Outcomes of Extracorporeal Shockwave Lithotripsy: A Systematic Review and Meta-analysis”. We have studied reviewers’comments carefully. According to the reviewers’ detailed suggestions, we have made a careful revision on the original manuscript. All revised portions are marked in the revised manuscript which we would like to submit for your kind consideration.** **We hope that the revisions in the manuscript and our accompanying responses will be sufficient to make our manuscript suitable for publication in Plos One. And wish you a happy Chinese Lunar New Year.**

**Kind regards.**

**Wang zheng-hao**

**E-mail: 614234687@qq.com**

**Corresponding author : Wang jia**

**E-mail address: Wangjia201707@163.com**

**Response to comments of editor**

1. The Table are indented in the text, please report them at the end of the article.

**Response:** We are very sorry to make the incorrect format and we corrected it according to requirement.

2. The first sentence of the Discussion should summarize the main results of the SR, please edit the sentence: “Following its implementation, SWL has since become the most frequently used approach for the treatment of urolithiasis (18).”

**Response:** It is a really good suggestion and we made the correction in this part,

3. The Discussion section is long and straight, please focus on the outcome of the study and shortened by 20%.

**Response:** Thanks for the comments and we have shortened this part according to the comments.

4. The paper related to the same topic that have been published in PLOS. Please consider to include: 1. Hai Wang, Libo Man, Guizhong Li, Guanglin Huang, Ning Liu, Jianwei Wang. Meta-Analysis of Stenting versus Non-Stenting for the Treatment of Ureteral Stones Research Article | published 09 Jan 2017 PLOS ONE.

**Response:** This is a very impressive study and we have cited this study in our article as the suggestion.

Special the thanks for your good comments

Wang zhenghao/Wang jia

2020-1-30
